# Grounding the data. A response to: Population finiteness is not a concern for null hypothesis significance testing when studying human behavior

**DOI:** 10.3389/fpsyg.2015.01169

**Published:** 2015-08-06

**Authors:** Thomas V. Pollet

**Affiliations:** Department of Experimental and Applied Psychology, VU University AmsterdamAmsterdam, Netherlands

**Keywords:** cross-cultural research, *p*-value, statistical inference, evolutionary psychology, ecological fallacy

I am thankful to Quillien (2015) for his response to my paper (Pollet, 2013), as it allows clarifying my position. Firstly, I would like to underline that the purpose of my paper was to flesh out the (implicit) statistical assumptions underpinning cross-cultural correlations. However, what I highlighted is but a side-issue when working with macro-level cross-cultural data (e.g., Poortinga, 1989; Mace and Pagel, 1994; Pollet et al., 2014). I would like to bring the discussion back to “earth” and clarify why I believe, in contrast to Quillien (2015), that finite populations might be problematic in this context.

I put forward that the sampling units for macro-level cross-cultural correlations are finite. In contrast, Quillien argues that this does not have to be the case. I believe for the examples I cited (Pollet, 2013: Table 1), the statistical data are clearly scores derived countries, states, etc. and are therefore by their (implicit) definition finite. Data at country/state/region level are very much unlike a population of people (Kuppens and Pollet, 2014) or an experiment where we can gather new, independent observations. Quillien presents no argument that these observed entities such as states/countries/etc. are in fact infinite but rather argues that the scores these researchers use represent something else.

Let us return to a specific example we both discussed, U.S. state scores (e.g., Kanazawa, 2006; Eppig et al., 2011). I put forward that if we sampled all possible units, here: all U.S. state scores, then there is no probability for the statistical population of U.S. states (for that point in time). The sample (U.S. States) matches the population we wish to make statistical inferences about (U.S. states). If the observations we sample are from a finite population (U.S. states), this needs to be corrected for (Pollet, 2013). Once we have sampled the last U.S. state, the population pool is empty: we sampled and measured everything. Not explicitly defining the “population” does not alter this, nor does assuming that these state scores are part of a larger, potentially infinite, whole. Many authors, such as Quillien, might want to make statistical or logical inferences beyond these scores, for example to other macro-level units or the “human mind,” but what we have in terms of data are U.S. state scores, plain and simple. These are by their very nature finite (fixed number of U.S. states). Quillien's argument thus seems to crucially rest on the claim that the observed unit of analysis is not a U.S. state score but rather something else. What this different unit of analysis would be is typically not clearly defined, neither by Quillien nor by the authors cited in Pollet (2013: Table 1). For now, like Quillien, let us assume the unit of analysis is some (aggregate) human social unit relevant to some evolutionary process. Such a stance, i.e., the data representing something else than a U.S. state score, is in my view deeply problematic. Firstly, if one assumes the scores are something else, then one needs to explicitly define the unit of analysis *a priori*, otherwise it seems reasonable that the unit of analysis is indeed a U.S. state score. Let us tentatively define this alternative unit of analysis as “a social unit in which humans live(d) relevant to an evolutionary process.” Why would a U.S. state then be representative of the pool of such social units in which humans lived? Perhaps these data can indeed tell us something about U.S. states, but it is unclear whether any documented statistical relationship would hold for other units fitting the broader definition. It is unclear whether any *statistical* inference can be done beyond U.S. states, as that is all we have. There is no logical reason to assume that any statistical relationship found for U.S. states should hold, for other “human social units,” such as for example: world regions, Canadian provinces, Polynesian chiefdoms, 19th century German states, hunter gatherer populations,…. Let alone that these different “human social units” can be meaningfully lumped together and assumed to be governed by the same evolutionary process, as Quillien seems to imply. In addition, suppose that we do follow Quillien's logic and pretend the pool is larger, and even infinite, then a different problem still arises: we have clearly drawn a biased sample (Good and Hardin, 2012). “Traditional” statistical inference based on rejecting a null hypothesis in the population cannot be applied in the first place as there was no random sampling. For example, Canadian provinces are underrepresented, absent even, in our sample, while they are in our statistical population.

Perhaps I am thus opposed to logical induction, and view moving from U.S. states to other not clearly defined “units” as problematic and Quillien does not. I will leave the reader to decide but it seems a much safer bet to stick to inferences about U.S. states, and not even rely on these data to make any inference on, for example, Canadian provinces. Ideally, researchers would then define and measure those, rather than assuming that one process must govern all these units.

Finally, if we take these state level data to be representative of processes at an *individual* level (“the human mind”), as several authors including Quillien seem to suggest, then the ecological fallacy looms: inferences from one level of statistical analysis need not correspond to a different level of analysis (see Robinson, 1950; Freedman, 1999; Kuppens and Pollet, 2014; Pollet et al., 2014). These arguments have been made at length elsewhere and will not be reiterated here.

In summary, in my view, the statistical inferences we can make based on units such as U.S. state scores can be about nothing else but U.S. states, as this is the *only* unit being sampled. One might want to strengthen the statistical and logical inference based on those data but this is likely invalid: either due to sampling bias and/or the ecological fallacy. I therefore maintain that the *p*-values commonly used for statistical inference are inappropriate for macro-level cross-cultural correlations when the sample matches the population closely.

## Conflict of interest statement

The author declares that the research was conducted in the absence of any commercial or financial relationships that could be construed as a potential conflict of interest.
